# Molecular Characterization of *Tinospora cordifolia* (Willd.) Miers Using Novel g-SSR Markers and Their Comparison with EST-SSR and SCoT Markers for Genetic Diversity Study

**DOI:** 10.3390/genes13112042

**Published:** 2022-11-05

**Authors:** Ritu Paliwal, Rakesh Singh, Debjani Roy Choudhury, Gunjan Tiwari, Ashok Kumar, K. C. Bhat, Rita Singh

**Affiliations:** 1 Division of Genomic Resources, ICAR-National Bureau of Plant Genetic Resources, New Delhi 110012, India; 2 Department of Biotechnology, School of Engineering and Technology, Sharda University, Greater Noida 201306, India; 3 Central Institute of Medicinal and Aromatic Planys, Lucknow 226015, India; 4 Division of Germplasm Evaluation, ICAR-National Bureau of Plant Genetic Resources, New Delhi 110012, India; 5 Division of Plant Exploration and Germplasm Collection, ICAR-National Bureau of Plant Genetic Resources, New Delhi 110012, India

**Keywords:** *Tinospora cordifolia*, genetic diversity, g-SSR, EST-SSR, SCoT markers, principal coordinate analysis (PCoA), cross-species transferability

## Abstract

In the present study, novel genomic-SSR (g-SSR) markers generated in our laboratory were used to characterize *Tinospora cordifolia* and related species. The g-SSR marker was also compared with EST-SSR and SCoT markers used earlier in our laboratory to assess the genetic diversity of *T. cordifolia*. A total of 26 accessions of *T. cordifolia* and 1 accession each of *Tinospora rumphii* and *Tinospora sinensis* were characterized using 65 novel g-SSR markers. A total of 125 alleles were detected with 49 polymorphic g-SSR markers. The number of alleles per locus varied from 1–4 with a mean value of 2.55 alleles per locus. Mean PIC, gene diversity, and heterozygosity were estimated to be 0.33, 0.41, and 0.65, respectively. The two species, namely *T. rumphii* and *T. sinensis*, showed cross-species transferability of g-SSRs developed in *T. cordifolia*. The success rate of cross-species transferability in *T. rumphii* was 95.3% and 93.8% in *T. sinensis*, proving the usefulness of this marker in genetic diversity studies of related species. The *Tinospora* accessions were also used for molecular characterization using SCoT and EST-SSR markers and compared for genetic diversity and cross-species transferability. The PIC, gene diversity, heterozygosity, and principal coordinate analysis showed that g-SSR is the better maker for a genetic diversity study of *T. cordifolia.* Additionally, high cross-species transferability of g-SSRs was found (95.3% and 93.8%) compared to EST-SSRs (68.8% and 67.7%) in *T. rumphii* and *T. sinensis*, respectively.

## 1. Introduction

Genetic diversity plays an important role in the stability of a species because it provides the required adaptation to the existing biotic and abiotic environmental conditions and enables change in the genetic composition to cope with the changes in the environment. There are various methods for evaluating genetic diversity in organisms including phenotypic, isozyme, biochemical, and molecular markers [1,2,3]. With the development of molecular biology, molecular markers have attained a special position. They are more authentic, less time-consuming, and allow the detailed analysis and evaluation of genetic diversity in plants. Among molecular markers, microsatellite markers are more reliable for a genetic diversity study because these markers are evenly distributed in the whole genome with tandem repeats of short nucleotides (1–6 bp) [4,5]. The SSRs are located in both the coding and non-coding regions of the genome and contribute a high level of polymorphism. They are multi-allelic, highly reproducible, and co-dominant in nature [6,7]. Hence, SSRs are highly advantageous, easy to assay, and have different applications like the construction of linkage maps, diversity assessment, and marker-assisted selection [8]. Start codon targeted markers (SCoT) are gene-targeted markers located in the translational start codon. This technique designs single primers from short, conserved regions flanking the ATG start codon. They are better markers than RAPD and ISSR and have gained importance in the study of genetic diversity analysis [9]. EST-SSR (expressed sequence tags microsatellite markers) has high intraspecific polymorphism, a co-dominant nature, and high reproducibility. They originate from genomic coding regions and directly reflect the diversity present in the genes. EST SSRs have been used to evaluate the genetic diversity of coconut [10] and *Dendrobium officinale* [11] and to construct core germplasm collection in olives [12].

*Tinospora cordifolia* (Willd.) Miers. is a diploid (2n = 22), large, glabrous, deciduous, climbing shrub that belongs to the family Menispermaceae. *T. cordifolia* usually requires fast-growing species like Jatropha, Moringa, and Neem (*Azadirachta indica*) for support to grow. *T. cordifolia* growing with Neem is known by the name “Neem Giloy”, and as it has many of the chemical properties of Neem and Giloe, it shows better therapeutic applications [13]. After the COVID-19 pandemic, many reports have shown the importance of Giloe in overcoming drug-induced liver injuries [14,15]. *T. Cordifolia* is well-known plant of deciduous and dry forests, dispersed in the tropical regions and above sea level from Kumaon to Assam, extending through West Bengal, Bihar, Deccan, Konkan, Karnataka, and Kerala. It is also found in Bangladesh, Sri Lanka, and China [16,17,18,19].

Due to the presence of enormous medicinal properties, this plant has been over-exploited by pharmaceutical companies and folk people for traditional remedies which led to the necessity of exploring more genetic information about this plant which would be useful in conservation. The literature survey shows that very little molecular characterization work has been performed so far on *T. cordifolia*. There are only a few reports available regarding the genetic diversity and phylogenetic study of *T. Cordifolia* using isozyme [20], ITS and cpDNA markers [21,22], SCoT markers [23], RAPD and ISSR markers [24,25,26,27], and SSR markers [28,29]. SSRs markers are excellent for linkage analysis, agronomic trait selection, and varietal identification. The present study has been done on the molecular characterization of *T. Cordifolia* using novel genomic SSR (g-SSR) markers developed in our laboratory [28] and is reported for the first time for the molecular characterization of *T. cordifolia*. Here, we are also reporting a comparison of the newly developed g-SSR with the EST-SSR markers developed in our laboratory [30] and SCoT markers [23] in deciphering the genetic diversity and population structure present in genus *Tinospora* along with cross-species transferability.

## 2. Materials and Methods

### 2.1. Plant Material and Genomic DNA Extraction

A total of 28 accessions which includes *T. cordifolia* (26 accessions), *T. rumphii* (one accession), and *T. sinensis* (one accession) cultivated and maintained at Issapur farms of ICAR-NBPGR, New Delhi, India, were selected (Table 1). Young leaves of these accessions were collected for genomic DNA extraction by using the modified CTAB method [31]. To remove phenolic compounds, 2% polyvinyl pyrrolidone (PVP) was added to the DNA extraction buffer. The quality of the DNA was checked on 0.8% agarose gel, and the quantity of DNA was estimated by a NanoDrop™ 1000 spectrophotometer (Thermo Fisher, Waltham, MA, USA). After quantification, the working concentration of each accession was made to 10 ng/µL and stored at 4 °C.

### 2.2. g-SSR PCR Amplification

Sixty-five g-SSR markers were used for the genetic diversity analysis of twenty-eight *Tinospora* accessions. All markers were generated by the “SSR enrichment method” in our laboratory [23] and primers were synthesized from Macrogen Inc. Out of 65 g-SSR primers, only 49 primer pairs were able to show good scorable bands on gel (Table S1). PCR reaction was performed in the total volume of 10 µL containing 3 µL genomic DNA (10 ng/µL), 1 µL of 10X buffer, 0.8 µL of 25 mM MgCl2, 0.3 µL of 10 mM dNTPs, 0.2 µL of each primer (10 nmol), 0.2 µL of Taq DNA polymerase enzyme (Fermentas, Life Sciences, City, Waltham, MS, USA), and 4.3 µL distilled water. PCR amplification was performed in a thermocycler using the following PCR cycle: initial denaturation at 94 °C for 4 min followed by 36 cycles of denaturation at 94 °C for the 30 s, annealing temperature (Ta °C) (Table S1) for 45 s, extension at 72 °C for 1 min, and final extension at 72 °C for 10 min. All amplified products were mixed with gel-loading dye (bromophenol blue, xylene cyanol, and sucrose) and then analyzed on 4% Metaphor agarose (Lonza, Basel, Switzerland) gel for 4 h at a constant supply of 120 V. Gel pictures were recorded using a Gel Documentation System (α Imager^®^, South San Francisco, CA, USA).

### 2.3. EST-SSR Amplification

A total of 96 EST-SSRs were used for the genetic diversity analysis of two additional *Tinospora* accessions (KCOP/41 and KCOP/18), one accession of *T. rumphii* (KCB/38), and one accession of *T. sinensis* (KC/NS/GD-42/14). The PCR amplification conditions were the same as Singh et al., 2016 [30]. All amplified products were mixed with gel-loading dye (bromophenol blue, xylene cyanol, and sucrose) and then analyzed on 4% Metaphor agarose (Lonza, Bend, OR, USA) gel for 4 h at a constant supply of 120 V. Gel pictures were recorded using a Gel Documentation System (α Imager^®^, USA).

### 2.4. SCoT Marker Amplification

Nineteen SCoT markers were used for the genetic diversity analysis of five additional *T. cordifolia* accession (IC-281970, IC-471321, IC-281968, KCOP/41, KCOP/18), one accession of *T. rumphii* (KCB/38), and one accession of *T. sinensis* (KC/NS/GD-42/14). The PCR amplification conditions were the same as Paliwal et al., 2013 [23]. All amplified products were mixed with gel-loading dye (bromophenol blue, xylene cyanol, and sucrose) and then analyzed on 1% agarose (Lonza, USA) gel for 4 h at a constant supply of 80 V. Gel pictures were recorded using a Gel Documentation System (α Imager^®^, USA).

### 2.5. Data Analysis

PCR products were scored visually on all 28 accessions of *Tinospora.* The band size of the amplified products was determined by comparison with size markers using a 50 bp DNA ladder (Fermentas, Life Sciences, USA). The genetic diversity assessment was done based on parameters like the number of alleles per locus, the major allele frequency, heterozygosity, gene diversity, and the polymorphic Information Content (PIC) for each locus using Power Marker V3.25 [32]. Genetic distances among all *Tinospora* accessions were calculated using Power Marker V3.25, and a Phylogenetic tree was constructed using a distance matrix in Power Marker and visualized using Figtree v 1.4.3 [33]. Populations were defined according to the states mentioned in Table 1. Delhi, Uttar Pradesh, and Haryana were considered three different populations, whereas Assam and Arunachal Pradesh were considered the fourth population (NE-North east). The model-based program STRUCTURE 2.3.4 [34] was used to infer the number of populations. The membership of each genotype was run for a range of genetic clusters from the value of K = 1 to K = 10. Each run was implemented over a burn-in period of 100,000 and 100,000 Monte Carlo Markov Chain replicates, the number of iterations being three for each population. Each K was then plotted to find the maximum peak of ΔK [35]. The online available “Structure harvester” program (http://taylor0.biology.ucla.edu (accessed on 15 July 2022)) was used to determine the final number of populations. The genetic relationship among *Tinospora* accessions was also analyzed by Principal Coordinate Analysis (PCoA) and Analysis of Molecular Variance (AMOVA) using GenAlEx V6.5 [36].

The genetic diversity of g-SSR markers was also compared with EST-SSR markers and SCoT markers for all 28 accessions. Hence, data scored across 28 accessions of *Tinospora* using 49 polymorphic g-SSRs, 80 polymorphic EST-SSRs [30], and 19 SCoT [23] markers were compared using different genetic parameters, i.e., the number of alleles per locus, heterozygosity, major allele frequency, polymorphic Information Content (PIC), and gene diversity for each locus using Power Marker 3.5 [32]. The genetic relatedness among 28 *Tinospora* accessions was also determined by the model-based program STRUCTURE and principal coordinate analysis (PCoA) generated by GenAlEx V6.5 [34] with 49 g-SSR, 80 EST-SSR, and 19 SCoT markers. Cross-transferability ability of g-SSRs and EST-SSRs was also compared in *T. rumphii* and *T. sinensis.*

## 3. Results

### 3.1. Diversity Assessment in Tinospora Species Using g-SSR

Sixty-five g-SSR markers were used to characterize twenty-eight accessions of *Tinospora* (26 accessions of *T. cordifolia*, 1 accession of *T. rumphii*, and 1 accession of *T. sinensis*). All 65 g-SSR markers were amplified by 26 *T. cordifolia* accessions, whereas, in the case of *T. rumphii*, 62 markers and 61 markers for *T. sinensis* were amplified. This shows that the cross-species transferability success rate was 95.3% in *T. rumphii* and 93.8% in *T. sinensis*. Out of these 65 markers used in the present study, 16 were monomorphic, whereas 49 g-SSR markers showed polymorphism. Only 49 polymorphic markers were used for the further study, which amplified a total of 125 alleles. The number of alleles per locus varied from 1 to 4 with a mean value of 2.55 alleles per locus. The maximum number of alleles (4) was amplified with primers TcgSSR15 and TcgSSR17, whereas 24 primers amplified 3 alleles and 22 primers amplified 2 alleles (Table S2). The *PIC* values ranged from 0.03 (TcgSSR-30 and TcgSSR-38) to 0.54 (TcgSSR-17) with a mean value of 0.33 (Table S2). The variability at each g-SSR locus was estimated in terms of major allele frequency which ranges from 0.46 (TcgSSR-17) to 1.00 (TcgSSR-43) with a mean value of 0.65 (Table S2). The heterozygosity varied from 0.04 to 1.00 with a mean value of 0.65. The gene diversity value for 49 polymorphic markers varied from 0.04 to 0.62 with a mean value of 0.41. TcgSSR-30 and TcgSSR-38 showed the minimum value (0.04), while TcgSSR17 (0.62) showed the maximum value of gene diversity (Table S2).

### 3.2. Genetic Relatedness Analysis

**Figure 1:**
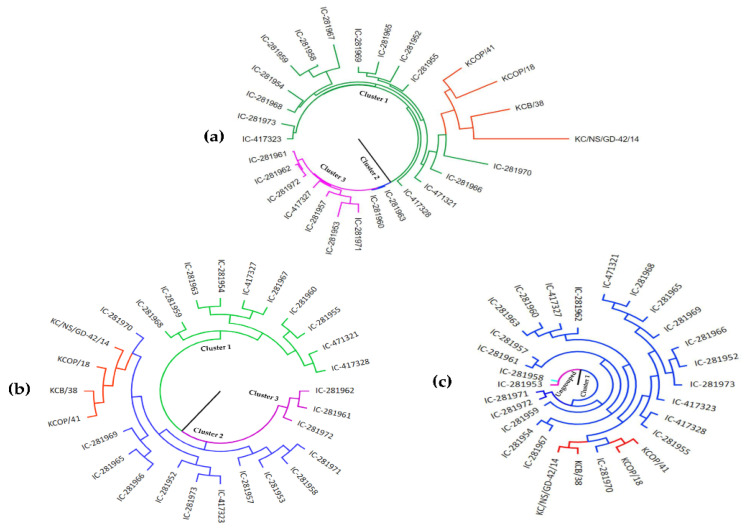
Genetic relationships study among 28 *Tinospora* accessions based on (**a**) g-SSR, (**b**) EST-SSR, and (**c**) SCoT markers.

Cluster analysis based on 49 g-SSR markers grouped 28 *Tinospora* accessions into 3 major clusters (Figure 1a). In cluster 1, nineteen accessions were grouped, in cluster 2 two accessions were grouped, and in cluster 3 seven accessions were grouped. In cluster 1, twelve from Delhi, three from Uttar Pradesh, two from Haryana, and one accession each from Assam (*T. rumphii*) and Arunachal Pradesh (*T. sinensis*) were present. *T. sinensis and T. rumphii* showed more similarity with two accessions of *T. cordifolia* (KCPO/18 and KCOP/41) from Haryana state. The accessions used in the present study were collected from three states (Delhi, Haryana, and Uttar Pradesh); however, location-specific grouping was not observed. The two species *T. rumphii* (KCB/38) and *T. sinensis* (KC/NS/GD-42/14) in cluster 1 showed that these two species share more similarities to each other than *T. cordifolia*. Phylogenetic analysis with EST-SSR markers (Figure 1b) showed three clusters. In clusters 1, 2, and 3, ten, fifteen, and three accessions were grouped, respectively. Seven accessions from Delhi and three accessions from Uttar Pradesh were present in cluster 1. Ten accessions from Delhi, two accessions from Haryana, one accession from Uttar Pradesh, one accession from Assam, and one accession from Arunachal Pradesh were present in cluster 2. Three accessions from Delhi were present in cluster 3. Cluster analysis with EST-SSR markers revealed that accessions KCOP/41 (Haryana), KCOP/18 (Haryana), KCB/38 (*T. rumphii*), KC/NS/GD-42/14 (*T. sinensis*), and IC-281970 from Delhi got grouped r (Cluster 2). Phylogenetic analysis with SCoT markers showed one cluster and two ungrouped accessions. Eighteen accessions from Delhi, four accessions from Uttar Pradesh, two accessions from Haryana, and one accession each from Assam and Arunachal Pradesh were present in cluster 1. The ungrouped accessions (IC-281958 and IC-281953) were from Delhi. With SCoT markers, (Figure 1c) the accessions KCOP/41 (Haryana), KCOP/18 (Haryana), KCB/38 (*T. rumphii*), KC/NS/GD-42/14 (*T. sinensis*), and IC-281970 (Delhi) grouped (Cluster 1). The comparison of the phylogenetic tree with all three-marker systems showed that *Tinospora* accessions KCOP/41 (Haryana), KCOP/18 (Haryana), KCB/38 (*T. rumphii*), S/GD-42/14 (*T. sinensis*), and IC-281970 share more similarity.

### 3.3. Population Structure Analysis

**Figure 2:**
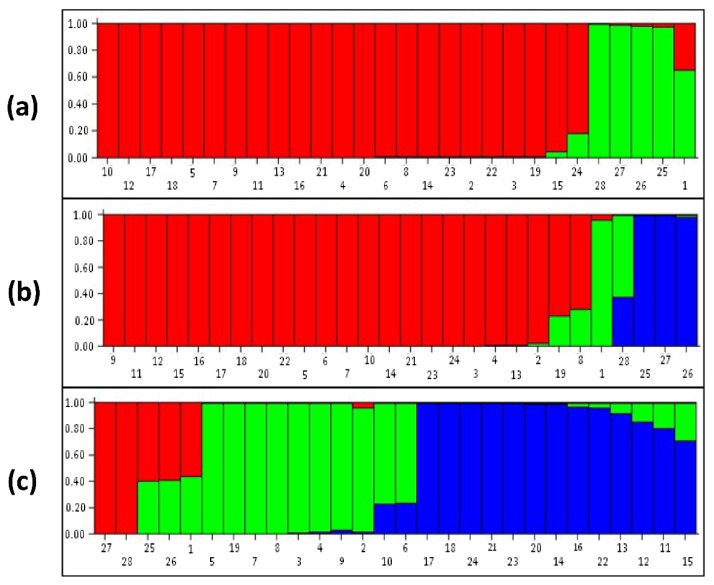
STRUCTURE bar plots of (**a**) g SSR (K = 2), (**b**) EST-SSR (K = 3), and (**c**) SCoT (K = 3) of 28 accessions of *Tinospora*.

Delta k was at the maximum at K = 2, and this was considered as the number of populations for 28 *Tinospora* accessions with g-SSR markers. In the structure analysis, different populations were categorized as pure or admixed. For categorization purposes, accessions with a score higher than 0.80 were considered pure, and those with a score lower than 0.80 as admixed (Figure 2a). Here, in population 1, there were 23 accessions categorized as pure, while in population 2 there were four accessions of which two were from other species T. *rumphii* (KCB/38) and *T. sinensis* (KC/NS/GD-42/14) along with two accessions of *T. cordifolia* (KCOP/41 and KCOP/18) from Haryana state. All these were pure except one *T. cordifolia* accession IC-281970 which was admixed (The numbers in the bar plot correspond to the serial no. of accessions given in Table 1). Delta k was the maximum at K = 3 for EST-SSR markers (Figure 2b). Population 1 had 23 accessions, population 2 had 2 accessions, and population 3 had 3 accessions. Population 1 had 21 pure and 2 admixed accessions. The two admixed were *T. cordifolia* accession IC-281968 from Delhi and IC-471321 accession from Uttar Pradesh. In population 2, *T. cordifolia* accession IC-281970 was pure and *T. sinensis* (KC/NS/GD-42/14) was considered admixed. In population 3, there were three accessions, KCOP/41, KCOP/18, and KCB/38 (*T. rumphii*), and all were considered pure. The population structure with SCoT markers revealed three populations (Figure 2c). Population 1 with five accessions showed accession KCB/38 (*T. rumphii*) and KC/NS/GD-42/14 (*T. sinensis*) were considered pure and accession KCOP/41, KCOP/18, and IC-281970 were in the admixed category. Population 2 had ten accessions with eight pure and two admixed. Population 3 had 13 accessions with 11 pure and 2 admixed. With EST-SSR markers, the *T. sinensis* and *T. rumphii* were separated into two different populations which were not observed with the g-SSR and SCoT markers.

### 3.4. Principal Coordinate and AMOVA Analysis

**Figure 3:**
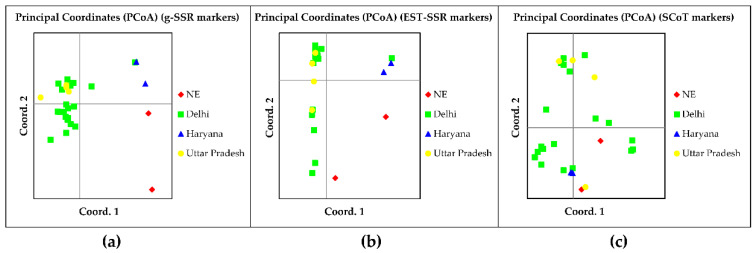
Principal Coordinate Analysis (PCoA) of 28 *Tinospora* accessions based on (**a**) g-SSR (**b**) EST-SSR (**c**) SCoT markers.

The principal coordinate analysis (PCoA) was performed with twenty-eight accessions considering the states as populations with all three marker systems. With g-SSR markers, axis-1 explained 16.97%, axis-2 explained 10.95%, and axis-3 explained 9.44% variation with the total percentage variation contributed by the first three axes being 37.37% (Table S4). This shows that a large diversity exists in the *Tinospora* species. The grouping pattern in the PCoA plot showed that *T. rumphii* (KCB/38) from Assam and *T. sinensis* (KC/NS/GD-42/14) from Arunachal Pradesh (NE (red), Figure 3a) were distinct from the other accessions of *T. cordifolia* and were more similar to each other. Among *T. cordifolia* accessions, KCOP/41 and KCOP/18 from Haryana (blue) and IC-281970 from Delhi were distinct, whereas other accessions showed more genetic relatedness to each other. The principal coordinate analysis (PCoA) performed with EST-SSR markers showed that axis-1 explained 26.84%, axis-2 explained 15.51%, and axis-3 explained 8.79% variation with the total percentage variation contributed by the first three axes being 51.13%, showing moderate genetic diversity among the accessions (Table S4). The distribution pattern in the PCoA plot showed *T. rumphii* (KCB/38) from Assam and *T. sinensis* (KC/NS/GD-42/14) from Arunachal Pradesh (NE (red) Figure 3b) being distinct and more similar to each other. Two *T. cordifolia* accessions KCOP/41 and KCOP/18 from Haryana (blue) were also found to be distinct. This result was similar to the PCoA plot pattern obtained by g-SSR markers, but unlike the pattern obtained in cluster analysis where all the above-mentioned accessions were grouped (Figure 1b). Principal coordinate analysis (PCoA) with SCoT markers axis 1 explained 26.65%, axis 2 explained 15.09%, and axis 3 explained 10.88% variation with total percentage variation contributed by the first three axes being 52.62% (Table S4), showing moderate genetic diversity among the accessions. The PCoA plot (Figure 3c) showed *T. rumphii* (KCB/38) from Assam and *T. sinensis* (KC/NS/GD-42/14) from Arunachal Pradesh (NE (red), Figure 3c) being mixed with other *Tinospora* accessions. Here, intermixing among the individual accessions was observed.

**Figure 4:**
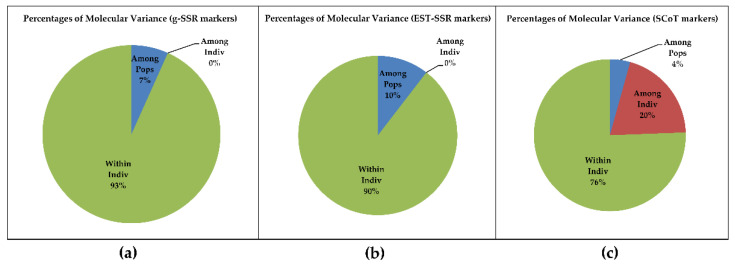
Analysis of Molecular variance (AMOVA) of 28 accessions of *Tinospora* based on (**a**) g-SSR (**b**) EST-SSR (**c**) SCoT markers.

AMOVA study was performed to estimate population differentiation among all *Tinospora* accessions. g-SSR data showed that 7% variations were present among the population, no variation among individuals, and 93% variations within the individuals (Figure 4a). Whereas, EST-SSR markers showed a 10% variation among the population, no variation among individuals, and 90% variation within individuals (Figure 4b). With SCoT markers there was a 4% variation among the population, 20% variation among individuals, and 76% variation within individuals (Figure 4c). AMOVA analysis indicates that EST-SSR markers are better markers to differentiate the *Tinospora* populations followed by the g-SSR marker system.

### 3.5. Comparison of g-SSR, EST-SSR and SCoT Markers in Genetic Diversity Study

**Figure 5:**
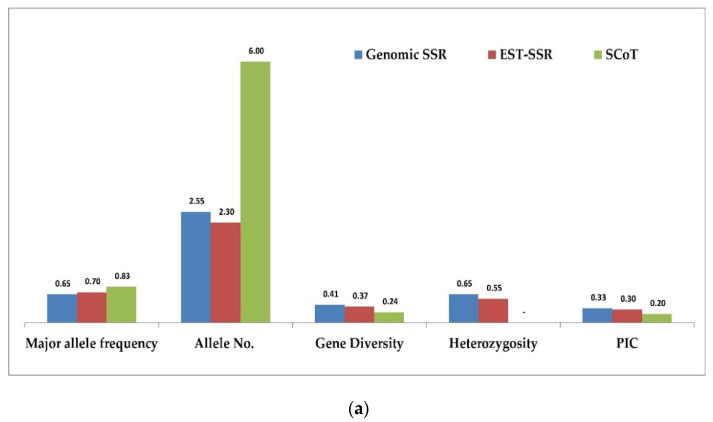

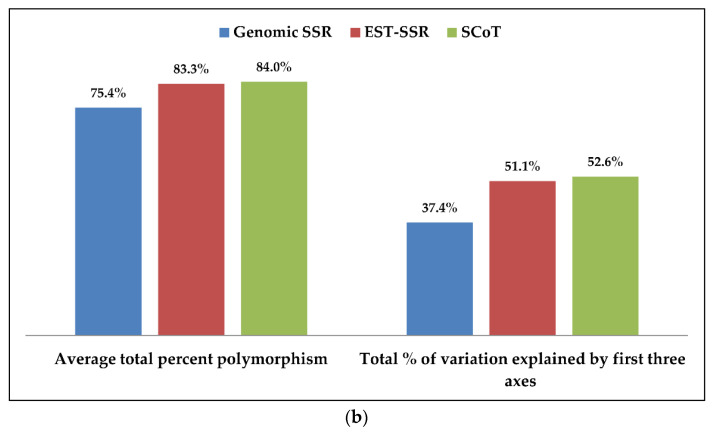
(**a**) Comparison of g-SSR, EST-SSR, and SCoT markers on diversity parameters of 28 *Tinospora* accessions. (**b**) Comparison of percent polymorphism between g-SSR, EST-SSR, and SCoT markers of 28 *Tinospora* accessions.

**Figure 6:**
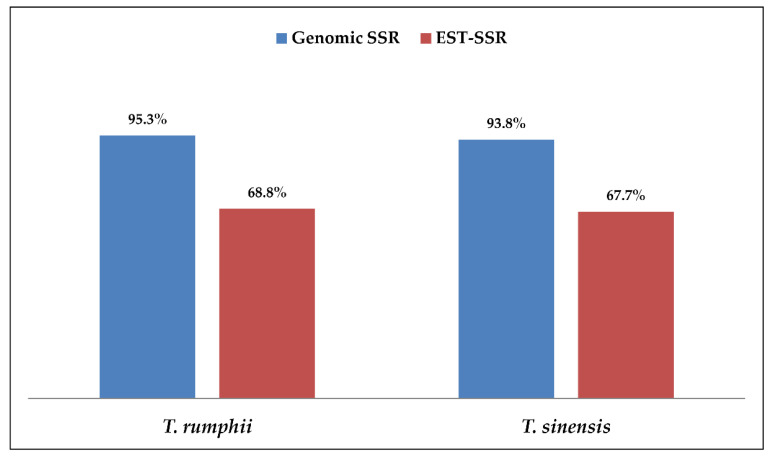
Comparison of cross-species transferability rate in *Tinospora rumphii* and *Tinospora sinensis* by using g-SSR and EST-SSR markers.

A comparison of 49 g-SSR, 80 EST-SSR, and 19 SCoT markers used for molecular characterization of 28 *Tinospora* accessions were made based on genetic diversity parameters, PCoA, AMOVA, and cross-species transferability level. The average number of bands produced per primer was compared, and it was found that in the case of g-SSRs, the average number of bands produced per primer was 2.55 while it was 2.30 and 6.0 for EST-SSRs and SCoT markers, respectively. The average major allele frequency estimated for g-SSRs, EST-SSRs, and SCoT were 0.65, 0.70, and 0.83, respectively (Tables S4 and S5 and Figure 5a). The heterozygosity varied from 0.04 to 1.00 with a mean value of 0.65 for g-SSRs and it ranged from 0.04 to 1.00 with a mean value of 0.55 for EST-SSR markers (Tables S2–S4 and Figure 5a). The gene diversity varied from 0.04 to 0.62 with a mean value of 0.41 for g-SSRs, it varied from 0.04 to 0.59 with a mean value of 0.37 for EST-SSR markers, while in SCoT markers it varied from 0.04 to 0.39 with a mean value of 0.24 (Tables S2–S4 and Figure 5a). The PIC values ranged from 0.04 to 0.54 with a mean value of 0.33 for g-SSRs, it ranged from 0.04 to 0.52 with a mean value of 0.30 for EST-SSR markers, while it varied from 0.04 to 0.30 with a mean value of 0.20 for SCoT markers (Tables S2–S4 and Figure 5a). Similarly, average per cent polymorphism was 75.4% in g-SSRs, while EST-SSRs and SCoT markers showed 83.3% and 84% average per cent polymorphic, respectively (Figure 5b). The cross-transferability success rate was found to be 95.3% in *T. rumphii* and 93.8% in *T. sinensis* with g-SSRs, while with EST-SSRs it was slightly lower, that is, 68.8% in *T. rumphii* and 67.7% in *T. sinensis* (Figure 6). PCoA analysis by g-SSR and EST-SSR presents *T. rumphii* and *T. sinensis* in separate coordinates with more similarity with each other, while the SCoT marker shows these species in the same coordinates but with more similarity with the accessions of Delhi and UP (Figure 3). AMOVA analysis shows that g-SSR gave a maximum variance of 93% within individuals, and SCoT markers gave a minimum variance of 76% within individuals. Among the population, the maximum variance was shown by EST-SSR markers, i.e., 10%, and the minimum by SCoT markers, i.e., 4%. There is no variance among individuals shown by g-SSR and EST-SSR. In contrast, SCoT markers show a 20% variance among individuals (Figure 4). The principal coordinate analysis based on 49 g-SSR data showed that the total percentage of variation explained by the first three axes was 37.4%, whereas analysis based on 80 EST-SSRs data explained 51.1% of the total, and, for analysis based on 19 SCoT markers, the total percentage variation explained by first three axes was 52.6% (Table S5 and Figure 5b).

## 4. Discussion

*T. cordifolia* is a plant of immense therapeutic value; therefore, it needs to be conserved. It is necessary to know about the total genetic diversity present in *T. cordifolia* from all over the country for its proper management and conservation. As there are very few reports available on the genetic diversity study of *T. cordifolia*, the present study attempts to characterize *T. cordifolia* using g-SSR markers. Twenty-six accessions of *T. cordifolia*, 1 accession of *T. rumphii*, and 1 accession of *T. sinensis* were characterized with 49 g-SSR markers. In our study, the number of alleles per locus varied from 1 to 4 with a mean value of 2.55 alleles per locus with g-SSR markers. In the case of *Paris polyphylla* [37], a medicinal plant, the number of alleles per locus ranged from 2 to 5. Similarly, average allele numbers have been reported in jute 2.2 [38], groundnut 2.5 [39], and sunflower 2.67 [40] using SSR markers, which is very similar to our report. According to Botstein et al. [41], the PIC value represents diversity within accessions and the degree of polymorphism at each locus. The Polymorphic Information Content (*PIC*) in *Tinospora* varied from 0.03 to 0.53 with a mean value of 0.33, which is less than the mean *PIC* observed in other medicinal plants. Cheng et al. [42] observed a PIC value greater than 0.7 in *Paeonia lactiflora*, a *PIC* value of 0.76 was observed in *Astragalus mongholicus* [43], and a value of more than 0.7 was seen in *Smilax brasiliensis* [44]. However, there are reports that showed low PIC values such as in the case of Groundnut (0.25) [39] and *Moringa* (0.15) [45]. The gene diversity varied from 0.04 to 0.62 with a mean value of 0.41. This value is more significant than the gene diversity reported in *Moringa* (0.18) [45], but higher gene diversity has been reported in the common bean (0.6) [46]. Heterozygosity ranged from 0.04 to 1.0 with a mean value of 0.66. A similar heterozygosity value was reported in *Astragalus mongholicus* ranging from 0.36 to 1.0 [47].

The principal coordinate analysis (PCoA) showed that the total percentage variation contributed by the first three axes was 37.40%, which shows a high diversity among the accessions. In the common bean *Phaseolus vulgaris*, the PCoA also showed a separation of 53% along coordinate 1 and 13% along coordinate 2 [46]. In the grass pea, the first two principal components explained 43.42% and 29.17% of the molecular variance, respectively [43]. Population Structure analysis also showed differentiation among *T. cordifolia*, *T. sinensis*, and *T. rumphii* study based on g-SSR markers. Similar studies were also reported in medicinal plants like *Andrographis paniculate* [48]. The genomic SSR markers developed in *T. cordifolia* were found transferable in the species *Tinospora rumphii* and *Tinospora sinensis*. Similarly, a study has been done on *Camellia oleifera* where SSRs markers showed a 90.4% transferability rate in *Camellia chekangoleosa* and 78.8% in *Camellia japonica* [49]. In another study on a tea plant (*Camellia sinensis*), a 100% SSR transferability rate was reported in cultivated *Camellia assamica* and *Camellia assamica* subsp. *Lasiocalyx* [50]. In peppermint (*Mentha piperita*), an 87% transferability rate of *Mentha arvensis* and 37% in *Mentha citrate* [51] were reported, and the soybean (*Glycine max*) showed a success rate of up to 85% in *Glycine clandestine* [52]. These studies show that there were different transferability rates based on the related species.

To compare g-SSR with EST-SSR markers which were earlier studied with 24 accessions, four new accessions were added using 80 EST-SSR markers. The revised analysis showed that the number of alleles generated was 2.30 alleles per locus. The cross-transferability success rate was 68.8% in *T. rumphii* and 67.7% in *T. sinensis*, which was lower than the transferability reported by the g-SSR makers (95.3% and 93.8%). Three populations (K = 3) were obtained with EST-SSR markers, and a similar population number was reported in *Monochasma savatieri* with EST-SSR markers [53]. The principal coordinate analysis (PCoA) with EST-SSR markers gave a total percentage variation contributed by the first three axes of 51.13%, showing good diversity among the accessions. The same type of percent variation has also been reported in the medicinal plant *Monochasma savatieri* using EST-SSR markers [53]. An AMOVA-based study in Tinospora by EST-SSRs showed 90% variation within individuals, which is higher than the medicinal plant *Panax vietnamensis* (63.17%) using EST-SSR markers [54]. It is observed that EST-SSR markers have been able to differentiate the *Tinospora* populations at a moderate level in comparison to g-SSR markers. This may be due to the generation of EST-SSR markers from more conserved and expressed gene sequences compared to genomic sequences which are present in the whole genome and have less or no selection pressure than expressed regions.

For comparison with g-SSR and SCoT markers, seven accessions were added using 19 ScoT markers. ScoT markers showed high polymorphism (84%) with a high average number of alleles per primer (6.0) reported. A study of the *Hedera helix* also showed high polymorphism (95.78%) with a low allele number (1.34) [55]. The PIC value with SCoT was 0.20, similar to that found in the *Hedera helix* [55]. The population structure analysis showed that SCoT markers could not differentiate the two species of *Tinospora* from *T. cordifolia* accessions from Delhi, UP states. Similar studies with SCoT markers showed pure and admixture individuals in medicinal plants like *Dendrobium* and *Crepidium* with three (K = 3) and two (K = 2) populations, respectively [56,57]. AMOVA analysis showed a 4% variation among the population which was observed at 34% in *Clerodendrum serratum* [58]. When gene diversity parameters were compared with *g-SSR*, *EST-SSR*, and *SCoT markers* on 28 *Tinospora* accessions, it was found that the major allele frequency is the lowest with g-SSRs (0.65) and the maximum in SCoT markers (0.83). The number of alleles per locus found was the maximum (6.0) with SCoT followed by g-SSRs (2.55), while it was the minimum with EST-SSR (2.30). The other parameters like heterozygosity (mean value of 0.65 for g-SSR and 0.55 for EST-SSR), gene diversity (mean value of 0.41 for g-SSR, 0.37 for EST-SSR, and 0.24 for SCoT), and *PIC* value (mean value of 0.35 for g-SSR, 0.30 for EST-SSR, and 0.20 for SCoT) showed that g-SSR markers were highly polymorphic compared to EST-SSRs and SCoT markers. In a similar type of study, high PIC was reported with EST-SSR (0.70) in comparison to g-SSR (0.63) in sugarcane [59], which is contradictory to our findings.

## 5. Conclusions

The genetic diversity assessment among *T. cordifolia* accessions with novel g-SSR markers revealed high genetic variation among accessions, and its comparison with EST-SSR and SCoT markers-based PIC, gene diversity, heterozygosity, and principal coordinate analysis supported the same conclusion when these parameters were compared among three markers systems (g-SSR, EST-SSR, and SCoT). In addition, the two species of genus *Tinospora* used in the present study, namely *T. rumphii* and *T. sinensis*, showed higher cross-species transferability of g-SSRs compared to EST-SSRs, which shows the usefulness of g-SSR markers in the genetic diversity study of *T. cordifolia* and related species. Thus, g-SSR markers developed in our laboratory were found to be highly polymorphic and powerful tools for genetic diversity analyses of the *Tinospora* germplasms.

## Figures and Tables

**Table 1:** Details of *Tinospora* accessions used in genetic diversity study.

S. No.	IC Number	Status	Source	District	State
1	IC-281970	Wild	Disturbed wild	Outer Delhi	Delhi
2	IC-281966	Wild	Disturbed wild	Central Delhi	Delhi
3	IC-281965	Wild	Disturbed wild	Central Delhi	Delhi
4	IC-281969	Wild	Disturbed wild	West Delhi	Delhi
5	IC-281952	Wild	Disturbed wild	Central Delhi	Delhi
6	IC-281973	Wild	Disturbed wild	West Delhi	Delhi
7	IC-417323	Wild	Natural wild	Balrampur	Uttar Pradesh
8	IC-471321	Wild	Disturbed wild	Balrampur	Uttar Pradesh
9	IC-281955	Wild	Natural wild	Central Delhi	Delhi
10	IC-417328	Wild	Natural wild	Faizabad	Uttar Pradesh
11	IC-281960	Wild	Disturbed wild	Outer Delhi	Delhi
12	IC-281963	Wild	Disturbed wild	Central Delhi	Delhi
13	IC-417327	Wild	Natural wild	Basti	Uttar Pradesh
14	IC-281967	Wild	Disturbed wild	West Delhi	Delhi
15	IC-281954	Wild	Disturbed wild	Central Delhi	Delhi
16	IC-281962	Wild	Disturbed wild	North Delhi	Delhi
17	IC-281961	Wild	Disturbed wild	North Delhi	Delhi
18	IC-281972	Wild	Disturbed wild	Outer Delhi	Delhi
19	IC-281968	Wild	Disturbed wild	West Delhi	Delhi
20	IC-281959	Wild	Disturbed wild	West Delhi	Delhi
21	IC-281958	Wild	Disturbed wild	North Delhi	Delhi
22	IC-281957	Wild	Disturbed wild	Central Delhi	Delhi
23	IC-281971	Wild	Disturbed wild	Outer Delhi	Delhi
24	IC-281953	Wild	Garden	Central Delhi	Delhi
25	KCOP/41	Wild	Garden	Sirsa	Haryana
26	KCOP/18	Wild	Disturbed wild	Mahendragarh	Haryana
27	KCB/38 ^a^	Wild	Disturbed wild	North Cachar Hills	Assam
28	KC/NS/GD-42/14 ^b^	Wild	Disturbed wild	East Siang	Arunachal Pradesh

IC-Indigenous collections, ^a^-Tinospora rumphii, ^b^-Tinospora sinensis.

## Data Availability

Not applicable.

## Supplementary Materials

The following supporting information can be downloaded at: https://www.mdpi.com/article/10.3390/genes13112042/s1. Table S1: Details of g-SSR primers used for characterization 28 Tinospora accessions. Table S2: Major allele frequency, Allele number, Gene diversity, Heterozygosity and PIC generated by 49 g-SSR in 28 *Tinospora accessions*. Table S3: Major allele frequency, Allele number, Gene diversity, Heterozygosity and PIC generated by 80 EST-SSR in 28 *Tinospora* accessions. Table S4: Major allele frequency, Allele number, Gene diversity, Heterozygosity and PIC generated by 19 SCoT markers in 28 *Tinospora* accessions. Table S5: Percentage of variation explained by the first 3 axes among the *Tinospora* accessions by g-SSR, EST-SSR and SCoT markers.
