# Supplementary material for: Molecular Characterization of Tinospora cordifolia (Willd.) Miers Using Novel g-SSR Markers and Their Comparison with EST-SSR and SCoT Markers for Genetic Diversity Study
Source: Genes (Basel). 2022 Nov 5;13(11):2042. doi: 10.3390/genes13112042 (PMC9690116; doi:10.3390/genes13112042)
Supplement: Supplementary file 1 [file genes-13-02042-s001.zip › genes-1942176-supplementary.pdf]

Table S1. Details of g-SSR primers used for characterization 28 *Tinospora* accessions

S. N.	Primer	Forward Primer Sequence (5'-3')	Reverse Primer Sequence (5'-3')	Repeat Motif	Ta (°C)	Expected Product Size in (bp)	NCBI Accession numbers
1	Tcgssr1	CTTCGCTCTACCGCCTCAT	AGCTTCGGATCCACGTTACA	(GTT)2	52	103bp	-
2	Tcgssr2*	TTCTGCGCACAAGAGAGAGA	TTCAGTGAACGACTCCAACG	(GA)10..(AG)8	52	241bp	KT384120
3	Tcgssr3*	TTGCTGGTGTGTGTGAGAGA	TTCATTGCTTTGTGCAGTCC	(AG)21	52	205bp	KT384121
4	Tcgssr4*	TGGAGCACTTTGACAGAGCA	AGATGTCAATCATGCCACCA	(GTG)2CA(GTG)2	51.6	211bp	-
5	Tcgssr5	CCAGCTTCTCCACTTCTTCG	TATGGGTGCGTCAAAACAGA	(CA)4AAC(CA)2	53	153bp	KT384122
6	Tcgssr6*	CCCAAGCTTCGCATCCTAT	ATCGGAACGACAAGTTCGAC	(TTTT)6	50	216bp	-
7	Tcgssr7*	CAAGCTTCGGATCGTTTGAT	CGCAAAGGAAGAAGAGGAAA	(CAG)2..(CAG)3	50.3	163bp	-
8	Tcgssr8	GAGCTGCACCCAAACAATTT	GCATAGGAATTCGGGGGTAT	(AG)3..(AG)2	51.6	182bp	-
9	Tcgssr9*	AGATGTCAATCATGCCACCA	TGGAGCACTTTGACAGAGCA	(ACAAC)2	52	211bp	-
10	Tcgssr10*	AGAAAACTAGGCCGCGAGTA	CTTCGGATCGGTGGATTTCT	(AC)3	50	182bp	-
11	Tcgssr11	GCTTCGAATCAAGGCATGAA	CCCAAGCTTCGCATCTTTA	(GT)4	50	150bp	-
12	Tcgssr12*	CTCAATGAGAGGGGGTTCAA	AGGTATATTTGGCGCTTGGA	(CCTA)3	50	220bp	-
13	Tcgssr13*	CCAAGCTTCGCATCAATTCT	GGATGAGCTTTTGACGAAGG	(CTT)3	50	174bp	-
14	Tcgssr14*	TATGGGTGCGTCAAAACAGA	CCAGCTTCTCCACTTCTTCG	(GAA)3(CTA)3(GAA)2	52	153bp	-
15	Tcgssr15*	TTGGGGATTTTGAGAGATTTG	CCGCATAATACTAATGAGCAAGG	(TGA)2G(TGA)2G(TGA)2	52	150bp	KT384123
16	Tcgssr16	GAACAGGGGATTCGAAACAG	GGATGCGCTCTCCTTTGTTA	(TTC)3	50	155bp	-
17	Tcgssr17*	AATTGGGAGAGAGAGAGAGAG	TTCTGCGTCCCTCCCTCT	(GA)25	53	239bp	KT384124
18	Tcgssr18*	CGTGCACACGAGAGAGAGAG	GCAACTCCGACGTCTTTTTC	(GA)26	53	228bp	KT384125
19	Tcgssr19	TCCTCCAAAATCATCGGAAG	GAGAGAGCTCACCGTTTTGG	(AAC)2AAT(AAC)2	52	194bp	-
20	Tcgssr20*	TTCTACTTCCTTCGCCTCCA	CACCAATCCACCCATTTTTC	(CT)18	52	171bp	KT384126
21	Tcgssr21*	TCAGACGAGAAACGAGCGTA	TCTTCACATTCGACGCAGAC	(AG)24	51.6	193bp	KT384128
22	Tcgssr22*	GAGGTTGTCCCACCAGCTTA	TTGATCCACTATGCGACCTG	(AAAA)4	52	234bp	-
23	Tcgssr23*	ACCCCAAGCTTCGCTAATTT	ACGTGTGTCGCTCAATCAAC	(GAGGT)3	52	151bp	-
24	Tcgssr24*	GCTTCGGACCCTATTCCTCT	CAGCTTAACCTGCAACACCA	(CTCT)4	50	183bp	-
25	Tcgssr25	GCGCAAGAAGACGGTAACA	GCTGCTGAGACGAGTTGTTG	(AAC)9(AAC)4AG (CAA)9(CAG)5	53	154bp	KT384129
26	Tcgssr26	AGCGATCGTGTTTGCACTTA	ATCAGCCACCACATTCACCT	(TATA)2	52	199bp	-
27	Tcgssr27*	GCTGTTGGGCAGTTCTTAGC	ACGAAATGGAAATGGGAATG	(CT)4..(CTCT)4	52	177bp	-
28	Tcgssr28*	CCATCTCACTGTGCGTGTCT	GCACGAGTGTGTGTGTGTGA	(TC)16	53	215bp	KT384130
29	Tcgssr29	GACCCCAAGCTTCGCTAAAT	ATGCAAAAACGCAGATAGGG	(GAA)3..(GAA)2	53	215bp	-

**30**	Tcgssr30*	TCTGATGTGTGTTGCGTGAA	GCTCCTTCCTTCATCATTCG	(TGC)2(TG)3(TGC)3(TG)3	53	161bp	KT384131
**31**	Tcgssr31*	TTGCTGTGCATCAATACCAAC	TGCAAAACACAACCACAACA	(GTGT)5	52	185bp	KT384132
**32**	Tcgssr32*	AGCTCCCTCTCTCACGCAAT	GTGCGAGATAAAGAGAGAAACTG	(TC)14	52	190bp	KT384133
**33**	Tcgssr33*	CGTGGTTTCTCGCAGCTCT	CAAGCTTCGCTACGAGAGAGA	(CT)8…(CT)7; (TC)7	52	348bp	-
**34**	Tcgssr34*	TGCAGTCTCTCGTCGCTCT	GCAAGAGAGAGCTTGCCAAA	(CT)6	52	241bp	-
**35**	Tcgssr35*	GCCTATCGGCGTAACAACAT	AAGGCAGACACGGTCCTAGA	(CTGCTG)4..(TC)4	52	234bp	KT384135
**36**	Tcgssr36*	CGATCCGAAGGCTGTGATTA	TGTGCTGAAATCTTGCCAAA	(AAAA)N	52	194bp	KT384136
**37**	Tcgssr37	TGTTGCATGTTTGAGGCATT	GCCCTGGTTCTTTCTTCCTT	(AAAA)N	52	227	KT384137
**38**	Tcgssr38*	AATGAACGCCAGCAACCTAC	GCATAGCAAGGCAGGGTAAG	(AAAA)N	52	154bp	KT384138
**39**	Tcgssr39*	ACCGCAGTCACACTCACAAC	GACAGGCATAGGGCAATGAT	(AGAG)N	52	237bp	KT384139
**40**	Tcgssr40*	GATGCGATCGAGGTTTATCC	GAAATTGGCTTGGAATTGGA	(AAAA)N	51.6	152bp	KT384140
**41**	Tcgssr41	CAACCCCCAGTAGTCAAGGA	TTCAGCGAAATAGGCTTGCT	(AAAA)N	52	235bp	KT384141
**42**	Tcgssr42*	GAGAGTTGCTGCCCTAGCTG	CCTTTCCTTAGGCGCAAAAT	(CTT)4..(CTT)2	52	178bp	KT384142
**43**	Tcgssr43*	CAACGTGTTCTCACCACACA	GGGTCACGATATCGGAAGAA	(TCTC)N..(TCTCTC)N	52	176bp	KT384143
**44**	Tcgssr44	TATTCGCCCTTTGCATTTTT	GCATATTGGGCACTGCTTCT	(A)10..(A)8..(A)4	52	208bp	KT384144
**45**	Tcgssr45	AAACAAAAGGTCACAAATCATCA	ATGACGCACAGCCTAATTGA	(AAT)5	52	152bp	KT384145
**46**	Tcgssr46*	TCAAATGAAGAACAGGGGAGA	CACACCAGCAGCTGCATAAC	(GT)5	52	153bp	KT384147
**47**	Tcgssr47*	AAGTTCCAGGGAGGAGGTGT	TTTGGGACGCAAAGTTTTCT	(CA)13A(AC)13A(AC)14	50.3	177bp	KT384148
**48**	Tcgssr48*	AGAACCCAGCAGTGATTTGC	TGCCTACGGGCTAGATCAAC	(AAAA)4	51.6	203bp	KT384149
**49**	Tcgssr49*	GATGAAATCCCGAGGACGAT	CCCCAAGCATAGGAAATTAGC	(AAAA)n(A)7	52	163bp	KT384150
**50**	Tcgssr50*	TTCGGGCAAAGATGAATAGG	GCAGCTGCATAACCCAGAAT	(GT)5	51.6	209bp	KT384151
**51**	Tcgssr51*	GGACATATGGCCAAGGACTG	TCAATGGCAAACACTCTTGC	(AAC)11GA(CAA)10	52	236bp	KT384091
**52**	Tcgssr52*	TAGGGACACCAGAGGCAAAT	ACCTTTGACAGCCGAAGTTG	(AAC)22	52	248bp	KT384092
**53**	Tcgssr53*	ACCGTCTCCATGCTTTTT GA	AAC ACA TAG CTT ATG CAA CA	(GT)16	49	206bp	KT384168
**54**	Tcgssr54*	CATGTGCTCGTGTGTGTGTG	CGCGTGCTTTCTCTCTCTCT	(GT)9..(AG)31	51.6	100bp	KT384084
**55**	Tcgssr55*	TCTCTCTCTCTCTCTGTGAGAT GTAAA	TCTTCGGTCTCTCCCTCTCC	(CT)12CA(CT)15	53	167bp	KT384087
**56**	Tcgssr56*	AATCTCTCTCTCTCTCTCTCT	GTCCCTCCCTCTTTCTCTGC	(CT)12CA(CT)15	53	147bp	KT384087
**57**	Tcgssr57*	GGTTGGATCGTCTTGATTCC	AACCATTGCAAGTTCTTCAAAA	(CT)23	53	151bp	KT384088
**58**	Tcgssr58*	AGAGAGAGAGAGAGAGAGAGA	CCA GTA CAC CCA CGT CAC TG	(GA)29	50.3	175bp	KT384099
**59**	Tcgssr59	TAGTCCCTTCGCCAATTGAT	CCC TCT CTC TCA GCC CTC TA	(GA)5..(GA)7..(GA)5	50.3	168bp	KT384110
**60**	Tcgssr60*	ATTCTCGTCGACCGATTCTG	TTTTGACACACAGCACACACA	(GA)15	51.6	195bp	KT384114

**61**	Tcgssr61	CAAATCTTGAGCGCAAACACT	TTGTAAGTGGTAATTAAGTGGGTTT T	(AG)28	50.3	163bp	KT384116
**62**	Tcgssr62*	TTGCCTAATAATGGCTGCAA	CTCTTGCTTACGCGTGGACT	(CA)3CC(CA)2A(CA)16	49	100bp	-
**63**	Tcgssr63*	TCCCATGTATCCTCCCCTTC	GACACGAACCAAGGATATAAGC	(GT)5..(GT)8	53	152bp	KT384160
**64**	Tcgssr64*	GAAAAACCAGTTGAGAGAG AGAGA	TGTCCATTCCTTTAAGCGTCA	(GA)25	51.6	152bp	KT384107
**65**	Tcgssr65	TGGGAACTGTTTCAATCAAATG	TTCAAAAAGAAGACTTAGTTGTCTC G	(CT)27 CC (CT)9	49	214bp	KT384083

*Polymorphic markers

Table S2. Major allele frequency, Allele number, Gene diversity, Heterozygosity and PIC generated by 49 g-SSR in 28 *Tinospora accessions*

Marker	Major Allele Frequency	Allele No	Gene Diversity	Heterozygosity	PIC
**Tcgssr17**	0.463	4	0.6173	0.8519	0.5399
**Tcgssr23**	0.4783	3	0.6115	0.913	0.5321
**Tcgssr14**	0.5357	3	0.574	0.9286	0.4913
**Tcgssr56**	0.4821	3	0.5759	0.9643	0.4839
**Tcgssr20**	0.5357	3	0.5644	0.8571	0.4769
**Tcgssr57**	0.5192	3	0.5614	0.9615	0.4683
**Tcgssr39**	0.5179	3	0.5453	0.9643	0.4442
**Tcgssr15**	0.5556	4	0.5384	0.8889	0.4435
**Tcgssr40**	0.5577	3	0.5377	0.8846	0.4426
**Tcgssr62**	0.6071	3	0.523	0.6786	0.442
**Tcgssr13**	0.6087	3	0.5189	0.5217	0.4361
**Tcgssr64**	0.5000	3	0.5397	0.913	0.4338
**Tcgssr55**	0.5000	3	0.5368	1.000	0.4295
**Tcgssr4**	0.4815	3	0.535	0.963	0.4262
**Tcgssr31**	0.5893	3	0.5108	0.8214	0.4119
**Tcgssr54**	0.5217	3	0.5189	0.8696	0.405
**Tcgssr2**	0.5000	3	0.5185	0.9615	0.4026
**Tcgssr10**	0.5000	3	0.5185	0.9231	0.4026
**Tcgssr49**	0.5000	3	0.5172	1.000	0.4007
**Tcgssr6**	0.5179	3	0.5159	0.9643	0.4000
**Tcgssr33**	0.5192	2	0.4993	0.8846	0.3746
**Tcgssr58**	0.5217	2	0.4991	0.6957	0.3745
**Tcgssr60**	0.5217	2	0.4991	0.8696	0.3745
**Tcgssr52**	0.5370	2	0.4973	0.9259	0.3736
**Tcgssr28**	0.5577	2	0.4933	0.8846	0.3716
**Tcgssr51**	0.5714	2	0.4898	0.8571	0.3698
**Tcgssr9**	0.5741	2	0.489	0.8519	0.3695
**Tcgssr44**	0.6607	3	0.4598	0.6786	0.3693
**Tcgssr34**	0.5893	2	0.4841	0.8214	0.3669
**Tcgssr47**	0.6042	2	0.4783	0.7083	0.3639
**Tcgssr35**	0.6071	2	0.477	0.7857	0.3633
**Tcgssr42**	0.6071	2	0.477	0.7857	0.3633
**Tcgssr63**	0.6346	2	0.4638	0.7308	0.3562
**Tcgssr27**	0.6429	2	0.4592	0.7143	0.3538
**Tcgssr53**	0.7037	3	0.4273	0.5185	0.3505
**Tcgssr50**	0.7143	2	0.4082	0.5714	0.3249
**Tcgssr3**	0.8036	3	0.3221	0.3929	0.2805
**Tcgssr24**	0.8036	2	0.3157	0.3929	0.2659
**Tcgssr12**	0.8393	2	0.2698	0.3214	0.2334
**Tcgssr21**	0.8929	3	0.1964	0.2143	0.1862

**Tcgssr22**	0.9107	3	0.1652	0.1429	0.1562
**Tcgssr18**	0.9286	3	0.1352	0.1429	0.1308
**Tcgssr36**	0.9286	2	0.1327	0.1429	0.1239
**Tcgssr48**	0.9423	2	0.1087	0.1154	0.1028
**Tcgssr32**	0.9643	2	0.0689	0.0714	0.0665
**Tcgssr7**	0.9792	2	0.0408	0.0417	0.0400
**Tcgssr30**	0.9821	2	0.0351	0.0357	0.0345
**Tcgssr38**	0.9821	2	0.0351	0.0357	0.0345
**Tcgssr43**	1.0000	1	0.0000	0.0000	0.0000
**Mean**	**0.653**	**2.551**	**0.4144**	**0.6564**	**0.3345**

Table S3. Major allele frequency, Allele number, Gene diversity, Heterozygosity and PIC generated by 80 EST-SSR in 28 *Tinospora* accessions

Marker	Major Allele Frequency	Allele No	Gene Diversity	Heterozygosity	PIC
**Tcestssr-3**	0.5714	2.0000	0.4898	0.8571	0.3698
**Tcestssr-4**	0.9286	2.0000	0.1327	0.0000	0.1239
**Tcestssr-5**	0.5769	2.0000	0.4882	0.8462	0.3690
**Tcestssr-6**	0.9464	2.0000	0.1014	0.0357	0.0963
**Tcestssr-7**	0.9643	2.0000	0.0689	0.0714	0.0665
**Tcestssr-8**	0.9444	2.0000	0.1049	0.1111	0.0994
**Tcestssr-9**	0.7679	2.0000	0.3565	0.4643	0.2930
**Tcestssr-11**	0.7143	2.0000	0.4082	0.5714	0.3249
**Tcestssr-12**	0.5179	2.0000	0.4994	0.9643	0.3747
**Tcestssr-13**	0.9600	2.0000	0.0768	0.0000	0.0739
**Tcestssr-14**	0.5357	2.0000	0.4974	0.9286	0.3737
**Tcestssr-15**	0.7200	2.0000	0.4032	0.5600	0.3219
**Tcestssr-16**	0.8600	2.0000	0.2408	0.0400	0.2118
**Tcestssr-17**	0.5357	2.0000	0.4974	0.7857	0.3737
**Tcestssr-18**	0.9200	2.0000	0.1472	0.1600	0.1364
**Tcestssr-19**	0.9000	2.0000	0.1800	0.2000	0.1638
**Tcestssr-20**	0.7143	3.0000	0.4177	0.5714	0.3441
**Tcestssr-21**	0.9107	2.0000	0.1626	0.1071	0.1494
**Tcestssr-22**	0.9286	2.0000	0.1327	0.0476	0.1239
**Tcestssr-23**	0.6875	2.0000	0.4297	0.6250	0.3374
**Tcestssr-26**	0.9643	2.0000	0.0689	0.0000	0.0665
**Tcestssr-29**	0.5536	3.0000	0.5364	0.8929	0.4391

**Tcestssr-30**	0.6481	2.0000	0.4561	0.7037	0.3521
**Tcestssr-32**	0.8929	3.0000	0.1971	0.2143	0.1879
**Tcestssr-33**	0.6111	2.0000	0.4753	0.7778	0.3623
**Tcestssr-34**	0.8929	2.0000	0.1913	0.2143	0.1730
**Tcestssr-36**	0.5000	3.0000	0.5800	1.0000	0.4918
**Tcestssr-37**	0.5556	2.0000	0.4938	0.8148	0.3719
**Tcestssr-38**	0.9423	2.0000	0.1087	0.1154	0.1028
**Tcestssr-39**	0.9800	2.0000	0.0392	0.0400	0.0384
**Tcestssr-41**	0.6481	2.0000	0.4561	0.7037	0.3521
**Tcestssr-42**	0.9815	2.0000	0.0364	0.0370	0.0357
**Tcestssr-44**	0.5536	4.0000	0.5886	0.6071	0.5220
**Tcestssr-45**	0.8750	2.0000	0.2188	0.0357	0.1948
**Tcestssr-46**	0.5000	2.0000	0.5000	0.6667	0.3750
**Tcestssr-47**	0.5714	2.0000	0.4898	0.8571	0.3698
**Tcestssr-48**	0.5179	2.0000	0.4994	0.9643	0.3747
**Tcestssr-49**	0.6786	3.0000	0.4471	0.6429	0.3618
**Tcestssr-50**	0.6875	4.0000	0.4627	0.4583	0.4013
**Tcestssr-51**	0.9231	2.0000	0.1420	0.1538	0.1319
**Tcestssr-52**	0.5000	3.0000	0.5343	1.0000	0.4259
**Tcestssr-53**	0.5741	2.0000	0.4890	0.7037	0.3695
**Tcestssr-54**	0.8696	2.0000	0.2268	0.1739	0.2011
**Tcestssr-55**	0.5536	3.0000	0.5096	0.8929	0.3967
**Tcestssr-56**	0.6296	2.0000	0.4664	0.7407	0.3576
**Tcestssr-57**	0.8214	2.0000	0.2934	0.0000	0.2503
**Tcestssr-58**	0.5185	2.0000	0.4993	0.9630	0.3747

**Tcestssr-59**	0.5536	3.0000	0.5236	0.8214	0.4190
**Tcestssr-60**	0.5179	2.0000	0.4994	0.8929	0.3747
**Tcestssr-61**	0.7105	2.0000	0.4114	0.5789	0.3267
**Tcestssr-62**	0.5893	2.0000	0.4841	0.8214	0.3669
**Tcestssr-63**	0.5000	4.0000	0.5658	0.7778	0.4728
**Tcestssr-65**	0.5714	2.0000	0.4898	0.8571	0.3698
**Tcestssr-66**	0.5536	2.0000	0.4943	0.8929	0.3721
**Tcestssr-68**	0.6200	2.0000	0.4712	0.6800	0.3602
**Tcestssr-70**	0.4630	3.0000	0.5919	0.8519	0.5044
**Tcestssr-71**	0.5357	2.0000	0.4974	0.8571	0.3737
**Tcestssr-72**	0.5370	2.0000	0.4973	0.9259	0.3736
**Tcestssr-73**	0.8333	2.0000	0.2778	0.0370	0.2392
**Tcestssr-74**	0.9464	2.0000	0.1014	0.1071	0.0963
**Tcestssr-75**	0.5000	3.0000	0.5733	0.8571	0.4823
**Tcestssr-76**	0.5893	3.0000	0.4981	0.8214	0.3906
**Tcestssr-77**	0.9815	2.0000	0.0364	0.0370	0.0357
**Tcestssr-78**	0.5000	3.0000	0.5478	1.0000	0.4456
**Tcestssr-79**	0.5357	2.0000	0.4974	0.9286	0.3737
**Tcestssr-80**	0.9821	2.0000	0.0351	0.0357	0.0345
**Tcestssr-81**	0.6964	2.0000	0.4228	0.6071	0.3334
**Tcestssr-82**	0.5714	3.0000	0.5045	0.8571	0.3940
**Tcestssr-83**	0.5000	3.0000	0.5178	1.0000	0.4016
**Tcestssr-84**	0.6071	2.0000	0.4770	0.7857	0.3633
**Tcestssr-85**	0.4815	4.0000	0.5521	0.8148	0.4512
**Tcestssr-86**	0.9815	2.0000	0.0364	0.0370	0.0357

**Tcestssr-87**	0.5357	3.0000	0.5644	0.8214	0.4769
**Tcestssr-89**	0.5000	2.0000	0.5000	1.0000	0.3750
**Tcestssr-90**	0.5185	2.0000	0.4993	0.8889	0.3747
**Tcestssr-91**	0.7407	2.0000	0.3841	0.5185	0.3103
**Tcestssr-92**	0.9821	2.0000	0.0351	0.0357	0.0345
**Tcestssr-94**	0.5000	3.0000	0.5478	0.9286	0.4456
**Tcestssr-95**	0.9821	2.0000	0.0351	0.0357	0.0345
**Tcestssr-96**	0.6071	2.0000	0.4770	0.7857	0.3633
**Mean**	**0.6972**	**2.3000**	**0.3686**	**0.5527**	**0.2952**

Table S4. Major allele frequency, Allele number, Gene diversity, Heterozygosity and PIC generated by 19 SCoT markers in 28 *Tinospora* accessions

Marker	Major Allele Frequency	Allele No	Gene Diversity	Heterozygosity	PIC
**SCoT1**	0.9643	6	0.0655	0	0.0606
**SCoT2**	0.7531	6	0.3182	0	0.2517
**SCoT3**	0.756	7	0.2902	0	0.2316
**SCoT4**	0.9	6	0.1778	0	0.1607
**SCoT5**	0.8571	6	0.2041	0	0.1686
**SCoT6**	0.7143	5	0.3327	0	0.2567
**SCoT7**	0.7407	6	0.3356	0	0.2673
**SCoT8**	0.8741	5	0.1679	0	0.1392
**SCoT9**	0.801	7	0.2992	0	0.2465
**SCoT10**	0.7884	7	0.2986	0	0.2446
**SCoT11**	0.9364	5	0.1033	0	0.0882
**SCoT12**	0.8365	4	0.1901	0	0.1504
**SCoT13**	0.9792	6	0.0399	0	0.0383
**SCoT14**	0.821	5	0.2725	0	0.2276
**SCoT15**	0.6646	7	0.39	0	0.2988
**SCoT16**	0.8636	7	0.2037	0	0.1726
**SCoT17**	0.8299	6	0.2643	0	0.2212
**SCoT18**	0.8571	7	0.2279	0	0.1931
**SCoT19**	0.74	6	0.3381	0	0.2675
**Mean**	**0.8251**	**6**	**0.2379**	**0**	**0.1939**

Table S5. Percentage of variation explained by the first 3 axes among the *Tinospora* accessions by g-SSR, EST-SSR and SCoT markers

Marker	Axis	%	Cum %
**g-SSR**	1	16.97	16.97
	2	10.95	27.93
	3	9.44	37.37
**EST-SSR**	1	26.84	26.84
	2	15.51	42.34
	3	8.79	51.13
**SCoT**	1	26.65	26.65
	2	15.09	41.74
	3	10.88	52.62
